# Influence of E-consultation on the Intention of First-Visit Patients to Select Medical Services: Results of a Scenario Survey

**DOI:** 10.2196/40993

**Published:** 2023-04-28

**Authors:** Miaojie Qi, Jiyu Cui, Xing Li, Youli Han

**Affiliations:** 1 Department of Health Management and Policy, School of Public Health, Capital Medical University Beijing China; 2 Office of Party Committee, Beijing Hospital of Traditional Chinese Medicine Beijing China

**Keywords:** e-consultation, medical selection, influence mechanism, scenario survey

## Abstract

**Background:**

E-consultation is expected to improve the information level of patients, affect patients’ subsequent judgments of medical services, and guide patients to make a reasonable medical selection in the future. Thus, it is important to understand the influence mechanism of e-consultation on patients’ medical selection.

**Objective:**

This study aims to explore the changes in first-visit patients’ understanding of disease and medical resources after e-consultation as well as the choice of follow-up medical services.

**Methods:**

Patients’ medical selection before and after e-consultation was compared using a scenario survey. Based on the service characteristics of the e-consultation platform, representative simulation scenarios were determined, and parallel control groups were set up considering the order effect in comparison. Finally, a total of 4 scenario simulation questionnaires were designed. A total of 4164 valid questionnaires were collected through the online questionnaire collection platform. Patients’ perception of disease severity, evaluation of treatment capacity of medical institutions, selection of hospitals and doctors, and other outcome indicators were tested to analyze the differences in patients’ evaluation and choice of medical services before and after e-consultation. Additionally, the results’ stability was tested by regression analysis.

**Results:**

In scenario 1 (mild case), before e-consultation, 14.1% (104/740) of participants considered their conditions as not serious. After e-consultation, 69.5% (539/775) of them considered their diseases as not serious. Furthermore, participants’ evaluation of the disease treatment capacity of medical institutions at all levels had improved after using e-consultation. In scenario 3 (severe case), before e-consultation, 54.1% (494/913) of the participants believed their diseases were very serious. After e-consultation, 16.6% (157/945) considered their diseases were very serious. The evaluation of disease treatment capacity of medical institutions in nontertiary hospitals decreased, whereas that of tertiary hospitals improved. In both mild and severe cases, before e-consultation, all of the participants were inclined to directly visit the hospital. After e-consultation, more than 71.4% (553/775) of the patients with mild diseases chose self-treatment, whereas those with severe diseases still opted for a face-to-face consultation. After e-consultation, patients who were set on being treated in a hospital, regardless of the disease severity, preferred to select the tertiary hospitals. Of the patients with mild diseases who chose to go to a hospital, 25.7% (57/222) wanted to consult online doctors face-to-face. By contrast, 56.4% (506/897) of the severe cases wanted to consult online doctors face-to-face.

**Conclusions:**

E-consultation can help patients accurately enhance their awareness of the disease and guide them to make a more reasonable medical selection. However, it is likely that e-consultation makes online medical services centralized. Additionally, the guiding effect of e-consultation is limited, and e-consultation needs to be combined with other supporting systems conducive to medical selection to play an improved role.

## Introduction

### Background

In China, e-consultation is a new type of health provision method that combines information technology and medical services. In this process, patients do not need to perform an online search for disease and treatment information. They only need to provide disease and symptom information to doctors, and then, the online doctors screen the information provided by the patients and make judgments via e-consultation [1]. Compared with non–real-time informational interactions in online medical communities, e-consultation enables patients to obtain the doctors’ response within a short time [2].

Although patients cannot undergo the necessary physical examination (eg, palpation) via e-consultation compared with face-to-face consultation, many studies have shown that there is no significant difference between online and offline face-to-face consultation in the diagnosis accuracy of common diseases and chronic diseases [3-6]. In China, online doctors are not allowed to make diagnoses for first-visit patients, but they could provide the disease information. Therefore, e-consultation is expected to considerably improve the information level of patients [7]. Decision-making theory believes that decision makers choose the largest expected utility selection after calculating the expected utility and its probability of different outcomes, with the calculations influenced by the external information. Therefore, patients can make secondary adjustments to medical decisions according to the changes in their disease information level [8]. Thus, this study aims to clarify whether e-consultation can affect patients’ medical choices.

At present, e-consultation in China is mainly provided by commercial internet platforms (eg, *Chunyuyisheng*, *Haodaifu*, and *Pinganhaoyisheng*). This made it challenging for us to effectively follow-up with the users and scrutinize their medical choices after e-consultation. Therefore, this study conducted a scenario survey to investigate the medical selection intention of patients after e-consultation. With the different scenarios set artificially, the participants made corresponding decisions that enabled us to compare the impact of different scenarios on the participants [9,10]. E-consultation was mainly operated through text, images, and other common online interactive chat forms. The information presentation mode of e-consultation can be simulated, and so a scenario survey is suitable for this research.

E-consultation users include first-visit and revisit patients. However, most online revisit patients are transferred from face-to-face consultation so that they can receive online follow-up disease management and other medical services provided by offline doctors [11,12]. E-consultation has now become an effective approach for offline doctors to provide service for revisiting patients. Further, as the revisiting patients would have chosen their preferred hospitals and doctors, influence mechanisms between revisiting and first-visit patients vary. However, the direct influence on medical selection forms the core of this study. Therefore, this study did not survey revisit patients, but it was rather designed to survey first-visit patients only.

### Theoretical Background

#### Unreasonable Medical Selection Under Information Asymmetry

Expected utility theory is the most basic and core paradigm in economics to study decision-making behaviors under uncertainty, and is applicable to all kinds of fields, including medical selection [13,14]. Under this theory, the decision makers will calculate the expected utility of various choices under uncertainty and finally choose the option with the highest utility. In medical selection, the utility was calculated according to the final gain (loss) after measurement of the health outcomes, economic cost, and time cost. Patients will select the best-utility hospital and doctors after calculating the expected utility of various medical choices [13,14]. High-grade hospitals impose higher medical costs but could treat more types of diseases. Therefore, high-grade hospitals have a greater utility for serious illnesses, whereas patients with mild diseases can be handled by lower-grade hospitals [15-17].

However, patients face insufficiency of medical information collection. On the one hand, the information barrier in hospitals is apparent, which makes it difficult for patients to obtain adequate information [18]. On the other hand, the health literacy of patients is limited, and so medical information cannot be the basis of effective decision making, even in the internet age of information explosion [19]. Patients are also unable to acquire comprehensive medical information, so they would rely on limited information to estimate the medical utility including reputation, social evaluation, and suggestions [20,21]. Concerning health outcomes, patients tend to value medical quality first, which leads them to select the highest-grade hospitals because they associate the good reputation of tertiary hospitals with better utility. Tertiary hospitals receive a large influx of patients, which not only reduces medical efficiency but also increases medical costs. Additionally, in the case of information asymmetry, the phenomenon of unreasonable medical selection is inevitable.

#### Advantageous Selection

Unreasonable medical selection occurs when low-risk patients select the medical services with a higher cost, which is a form of adverse selection. However, many studies have shown that people can also make appropriate choices under information asymmetry, which is called positive selection [22]. Positive selection is different from risk attitude (preference) and risk perception (belief). The core idea is that people have different understandings and perceptions of their own health risks, which leads to distinction in demand for market services, thus avoiding adverse selection.

Risk attitude refers to an individual’s degree of risk tolerance [23]. Patients with high-risk tolerance are more likely to choose primary medical institutions and take the risk of utilizing their health services, whereas patients with low-risk tolerance tend to choose high-level medical institutions. Therefore, the impact of patients’ risk attitudes should not be ignored in medical choice research.

Risk perception is the decision-makers’ judgment on the possibility of risk occurrence [24]. The judgment of the risk probability is derived from comprehension based on the decision-makers’ knowledge and experience, instead of the rigorous logical calculation. If a decision maker underestimates the probability of risk, overconfidence occurs. On the contrary, if a decision maker overestimates the probability of risk, overreaction occurs. In the scenario of medical choice, risk perception is reflected in patients’ understanding of the disease and the level of medical institutions. Different groups have different perceptions of the severity and process of the same risk [25].

#### Andersen’s Behavioral Model of Health Services Use

There are a number of factors that affect patients’ medical selection, and the influence of e-consultation on patients’ medical choices can be scientifically obtained only after controlling for the other influencing factors. Andersen’s Behavioral Model of Health Services Use absorbs the subjective and objective factors of patients, hospitals, doctors, and other aspects in the process of medical behavior, and has become one of the most representative models to explain medical behavior. With its constant adjustments, the model was most cited in the research of individual medical choice, medical expenditure, disease screening, drug use, and other medical and health service utilization behaviors [26]. Based on the Andersen’s model, this study selected and controlled the influencing factors of medical choice during e-consultation. Andersen’s model consists of 4 major parts: contextual characteristics, individual characteristics, health behaviors, and health outcomes. Contextual characteristics include circumstances and the environment; individual characteristics are determined by a person’s life circumstances, for example, genetics and socialization; health behaviors are an individual’s personal practices; and health outcomes include an individual’s health status and consumer satisfaction [26].

Andersen’s model is constructed for the whole process of medical services, so it is too complex and needs to be simplified according to research needs. This study is aimed at first-visit patients. In China, online doctors are only allowed to provide medical suggestions and information for first-visit patients, and providing diagnoses is illegal. Therefore, the health outcomes and 2 factors of health behavior (use of personal health services and process of medical care) were excluded. Hence, the factors influencing first-visit patients’ medical choices in this research were demography, organization, social structure, health beliefs, financing, and perceived and personal health practices. The factors selected in this study are shown in Table 1.

**Table 1:** Influencing factors of first-visit patients’ medical choice.

Subcomponents of Andersen’s model	Factors selected in this study
Demography	Gender, age
Organization	Accessibility of health care
Social structure	Education, profession, residence
Beliefs	Health literacy
Financing	Income, medical insurance
Perceived health practice	Risk perception
Personal health practice	Risk attitude

### Research Model and Hypotheses

According the true e-consultation record offered by the Haodf platform [27] and experience of doctors interviewed, the e-consultation process was summarized. E-consultation is a process of interactive communication between doctors and patients. In e-consultation, disease symptom description is fulfilled by patients, and information judgment and medical advice are offered by online doctors. If the patients’ description is inaccurate, the online doctors will repeat their questions and guide patients in providing the correct information. In the whole process of e-consultation, there are positive interactions between the doctors and patients. The disease judgments and treatment judgments are proposed after such doctor-patient communication, which greatly improves the patients’ participation and satisfaction. Additionally, during e-consultation, the accuracy of doctors’ judgments of disease will be highly improved, and the treatment suggestion offered by e-consultation becomes more targeted to the disease. Therefore, patients are more likely to trust and accept the diagnosis and treatment suggestions provided by online doctors. The following hypothesis is proposed in this study.

*Hypothesis 1:* Patients have a high degree of trust and acceptance in the online doctors’ disease judgments and advice.

The suggestions provided by the online doctors for first-visit patients can be divided into 4 categories: (1) disease diagnosis and treatment plan, (2) future disease visit and management plan, (3) further medical examination, and (4) other information related to the disease [28]. The online doctors provide the first suggestion for patients, indicating that the patients can be cured by self-treatment. If the online doctors decide that the patients need further medical examination, they will first provide a preliminary judgment and management plan and then recommend that the patients should be treated in the hospital directly. In conclusion, patients have access to diagnoses and treatment suggestions regardless of the severity of the disease.

Before e-consultation, patients should evaluate their disease situation and the treatment capacity of various medical institutions to determine which medical institutions to select. According to the theory of unreasonable medical selection, patients often overestimate the severity of their diseases and prefer to choose high-level hospitals to avoid medical risks to the greatest extent possible. After e-consultation, patients acquire adequate personal health information based on doctors’ judgments and treatment suggestions. They would then have a further understanding of their diseases and would reassess the disease severity more accurately. Eventually, more patients will come to believe that primary health services could meet their health demands. Therefore, this study proposes the following hypotheses.

*Hypothesis 2:* After e-consultation, patients assess the severity of their diseases more reasonably.

*Hypothesis 3:* After e-consultation, patients assess the treatment capacity of various medical institutions more reasonably.

For many patients with mild diseases, the doctors would suggest self-treatment. Once the patients have accepted the online doctors’ suggestion, those who originally preferred being treated in the hospital will reconsider and choose self-treatment. For patients with severe diseases, doctors would recommend that they be treated in the hospital directly, implying that their illnesses are critical. Accordingly, the preference for directly visiting the hospital will be justified, resulting in more patients visiting high-level hospitals. Therefore, the following hypothesis is proposed in this study.

*Hypothesis 4:* After e-consultation, patients’ choice of hospitals and doctors will change.

### Study Aim

The objective of this study was to explore the changes in first-visit patients’ understanding of diseases and medical resources after e-consultation and to determine the influence of e-consultation on the first-visit patients’ medical selection.

## Methods

### Study Design

#### Select Scientific Scenario

First, many studies have shown that under the disease condition suitable for e-consultation, the accuracy of e-consultation remains very high, reaching more than 90%; further, there is no substantial difference between e-consultation and offline consultation [4,5]. Second, the purpose of this study was to explore the choice of first-visit patients after using e-consultation, rather than the choice of the whole medical process. The influence of wrong medical suggestion will not be reflected in this study, because the patients need another consultation to ensure the online suggestions were incorrect. Based on these considerations, this study only considers the scenario of correct online suggestion.

Based on the data collected from hospitals in Beijing through the online platform Haodaifu, which is one of the leading e-consulting platforms in terms of the volume of doctors, patients, service quantity, service quality, reputation, among others, it was found that tertiary hospitals and chief physicians were the first choices of patients using e-consultation [29]. Therefore, this study only chose the tertiary hospitals and chief physicians as the simulation health service providers instead of cross-combining all levels of medical institutions and doctors. After analyzing the service volume of the departments in the online platform, it was found that the dermatology department was one of the departments with the highest service demands [30]. Additionally, in the previous interviews, the administrators and doctors from 6 tertiary hospitals believed that the dermatology department was representative in e-consultation. Therefore, dermatological diseases were selected as the simulated disease.

The simulated dermatological diseases were determined through expert interviews and face-to-face consultation. Dermatologists from top tertiary skin hospitals and with rich experience in e-consultation were selected, and the research design was meticulously introduced to them. After multiple rounds of group discussions, drug eruption was selected. First, drug eruption is the most common disease among daily e-consulted dermatological diseases, and so it was a suitable representative. Second, drug eruption has various disease types that encompass all severity levels. Hence, drug eruption was realizable to be set in different scenarios of disease status. Finally, from the patients’ point of view, the symptoms of different types of drug eruptions are similar. The main symptoms are erythema, urticaria, fever, and peeling skin. Therefore, selecting drug eruptions as simulated diseases can better reduce systematic bias because the common symptom descriptions can avoid differences in participants’ descriptions between scenarios.

**Figure 1:**
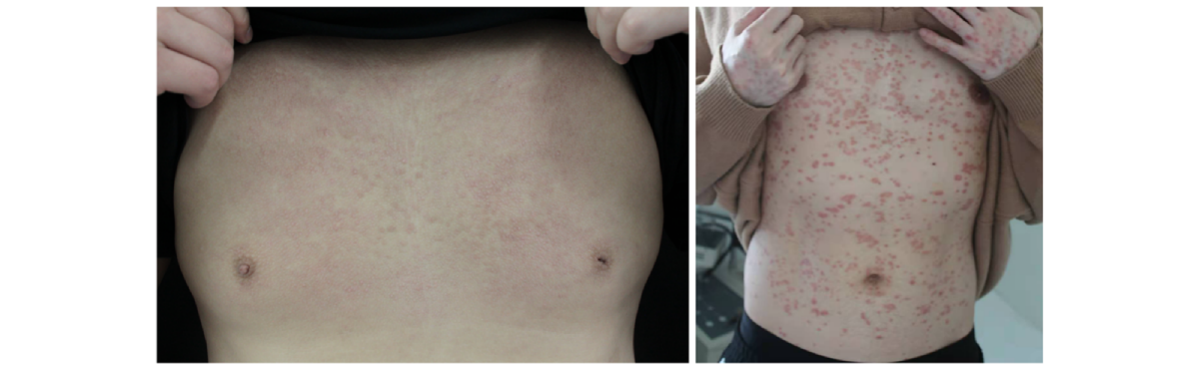
Image of disease symptom.

**Figure 2:**
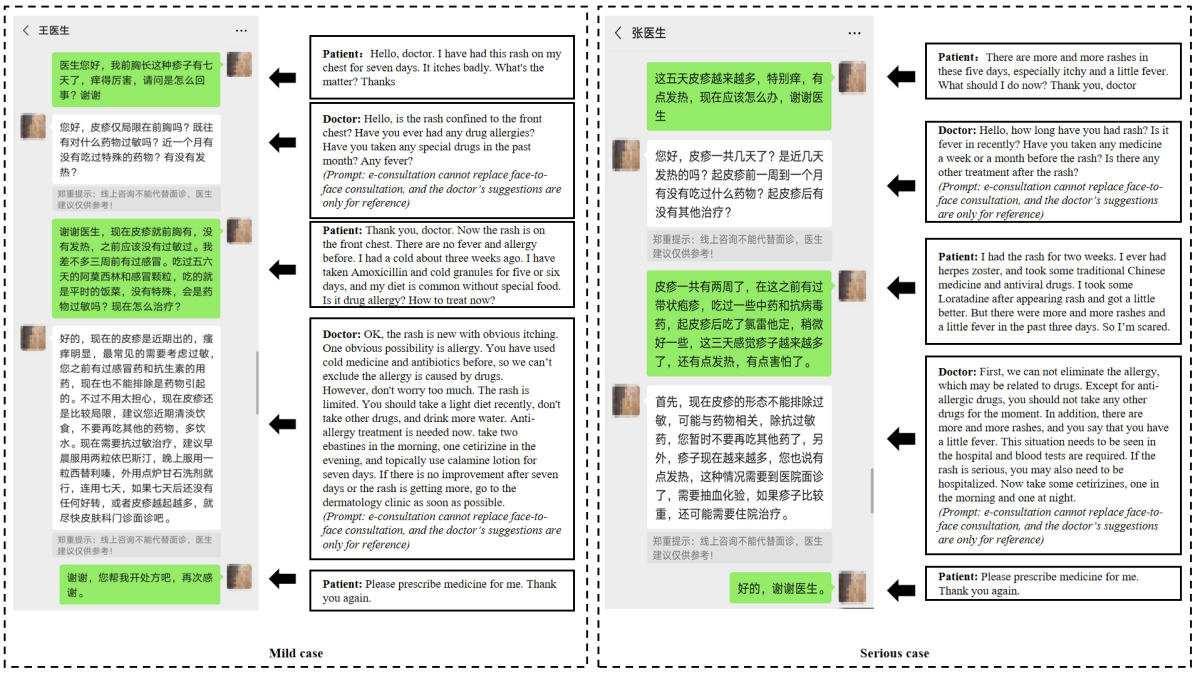
Sample e-consultation.

To determine the influence of the disease severity, both mild and severe cases were examined. Each case contained 2 figures: one is an image of symptoms of drug eruption, and the other is a screenshot of doctor-patient online communication regarding the disease. The symptom and communication images were adapted from real-world cases. In the case of mild drug eruption, the doctors provided the disease diagnosis and self-treatment suggestions. In the case of severe drug eruption, the doctors recommended that the patients should be treated in the hospital directly. See Figures 1 and 2 for the details of disease symptoms and e-consultation.

#### Scale Application in Scenario Survey

##### The Brief Health Literacy Screen

According to Andersen’s model, health literacy is a key factor in medical selection. However, common health literacy scales were too complex and needed a long response time. Therefore, scholars began to develop a simplified version of the Health Literacy Scale. After the test of explanatory power, 3 items were selected from 12 items and the Brief Health Literacy Screen (BHLS) was formed, which greatly reduced the number of items while ensuring sufficient effectiveness. Therefore, the BHLS was utilized in this study [31]. There were a total of 3 questions in the BHLS. The first 2 questions were “How often do you have someone help you read hospital materials?” and “How often do you have problems learning about your medical condition because of difficulty understanding written materials?” The answer choices for both questions were “always,” “often,” “sometimes,” “occasionally,” and “never.” The third question was “How confident are you filling out forms by yourself?,” and the answers were “extremely,” “quite a bit,” “somewhat,” “a little bit,” and “not at all.”

##### Risk Attitude

The Domain-Specific Risk-Taking (DOSPERT) Scale can measure risk attitude [23]. With the development of the questionnaire, the DOSPERT scale has been gradually simplified from 40 to 30 items and allows making effective choices according to the needs of the research field [32]. The items of the DOSPERT scale are divided into 5 domains (Ethical, Financial, Health/Safety, Recreational, and Social) and there were significant differences among the 5 domains. As this was a health-related study, the items of Health/Safety domain were classified. In this study, a 5-point Likert scale was selected, which had the following items: “Drinking heavily at a social function,” “Engaging in unprotected sex,” “Driving a car without wearing a seat belt,” “Riding a motorcycle without a helmet,” “Sunbathing without sunscreen,” and “Walking home alone at night in an unsafe area of town.” The respondents were asked “What do you think was the risk level of the following scenarios?,” with the options being “1=extremely,” “2=risky,” “3=not sure,” “4=slightly risky,” and “5=not at all risky.”

##### Risk Perception

Based on the idea of task completion probability assessment, this study simplified the task into disease severity and medical institution capability to reflect the risk perception. At different stages of the survey, the participants were asked to assess the severity of the disease and the probability of curing the disease in different medical institutions. The question on disease severity assessment was “How serious do you think is your disease?” and the answers were “not serious at all,” “not serious,” “unclear,” “serious,” and “very serious.”

#### Design the Questionnaires of Scenario Survey

After the simulation choice (drug eruption) was determined, a scenario survey was designed. The questionnaire was divided into 4 parts: the first part was the survey of sociodemographic characteristics, health literacy, and risk attitude of the respondents. The second part included the scenario perception of disease symptoms and intention survey of medical selection. After completing the first part, participants proceeded to a separate page and viewed a symptom image with the symptom description text. The mild drug eruption was named scenario 1, and the serious drug eruption was named scenario 2. The participants were then required to answer the questions about the perception of disease severity, probability of curing the disease, and medical selection: “Do you need to go directly to the hospital and what kind of doctor would you choose after getting this disease.” The third part contained the scenario perception of e-consultation and intention survey of medical selection. The respondent received e-consultation information and answered the questions about the perception of disease severity and selection of medical treatment again. The fourth part was the survey regarding the practical experience on drug eruption e-consultation. After completing the previous 3 parts of the questionnaire, all participants would be asked whether they have experienced drug eruption and whether they have received e-consultation after the eruption. Only those participants with practical experience were required to take the fourth part of the questionnaire.

**Figure 3:**
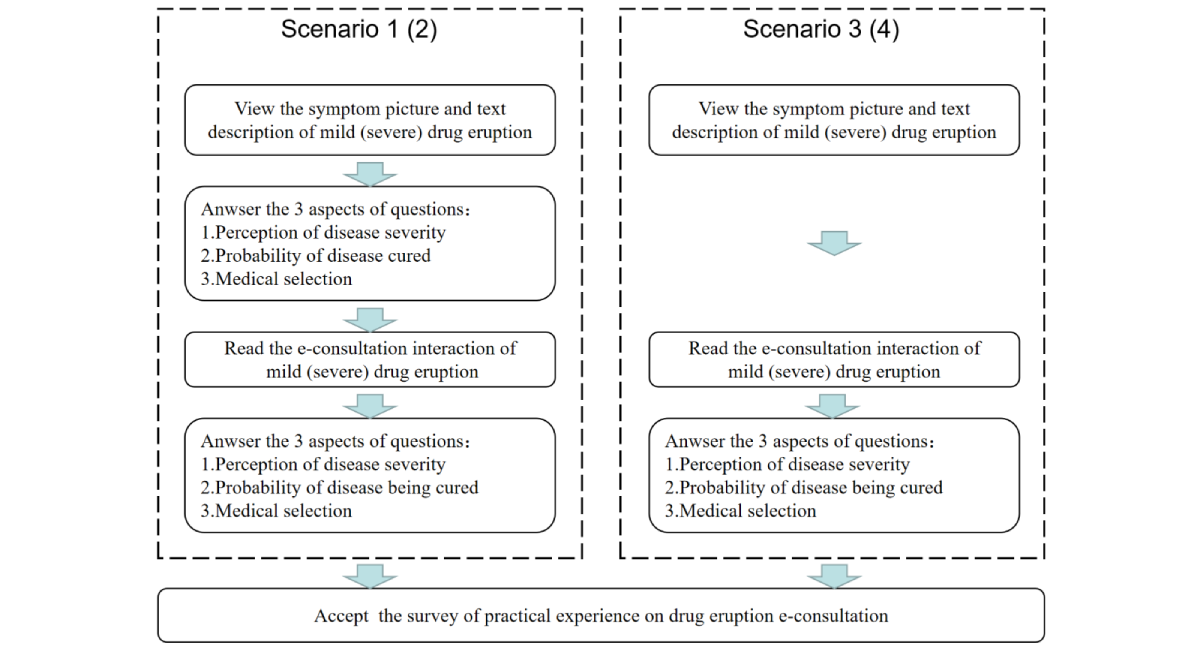
Flowchart of the 4 questionnaires.

As the participants needed to answer about their perception of disease severity and medical selection 2 times in 1 questionnaire, which may lead to anchoring effect and order effect, 2 control questionnaires were designed. In the 2 control questionnaires with the same scenario, participants were only required to provide answers after viewing the e-consultation process; the 2 control questionnaires were named scenarios 3 and 4. The distinction between the 4 questionnaires and the flowchart are shown in Figure 3.

### Data Collection

As doctors have accurate judgments about drug eruptions and rich medical resources for treatment, they were excluded as participants receiving e-consultation in this study. Besides, a previous study [33] has shown that there was no significant difference in the path coefficient of the factors influencing the public’s intention to use e-consultation, irrespective of whether the public used e-consultation or not. Therefore, this study only included participants with e-consultation experience. Those with practical experience of receiving e-consultation for drug eruption could better reflect the real medical choice; besides, it was important to verify the results of the scenario survey, and therefore these individuals were included this group. However, their practical experience would affect causality between research interventions and outcomes. Therefore, the data on participants without practical experience were collected for an analysis of the summary statistic, perception of disease and medical institution, intention of medical selection, and robustness test. The data on practical experience have been analyzed separately.

The survey was sent through the Wenjuanxing survey tool [34]. Before filling the electronic questionnaire, participants would be asked if they were a doctor. Once they answered “yes,” they would be forced to withdraw. For the data collection process within the platform, please refer to a previous study [33]. The platform provided the function of randomizing scenarios, so the participants could randomly complete 1 of the 4 questionnaires to reduce the selection bias and control the influence of basic characteristic on medical selection. To ensure the participants were fully involved in the scenario, the minimum filling time limit was set for each part of the questionnaire. The limit-time setting standard was based on the actual filling test: the limit time of the first part was 65 seconds, that of the second part was 25 seconds, that of the third part was 60 seconds, and that of the fourth part was 15 seconds. If the participants had practical experience with drug eruption e-consultation, the limit time of the fourth part was 35 seconds.

In addition to the quality control questions designed by the platform, we designed special quality control questions. In this study, the simulated doctors’ judgments were taken as the quality control standard, and a quality control question was set in the second part of the questionnaire: “In this consultation, doctors’ suggestions are: self-treatment or treated in hospital directly.” The participants assigned to scenarios 1 and 3 should choose “self-treatment,” and those assigned to scenarios 2 and 4 should choose “treated in hospital directly.” Once the participants made the wrong choice, their responses were considered invalid and were therefore removed. Finally, 607 questionnaires were excluded from the mild cases and 157 from the severe cases.

**Figure 4:**
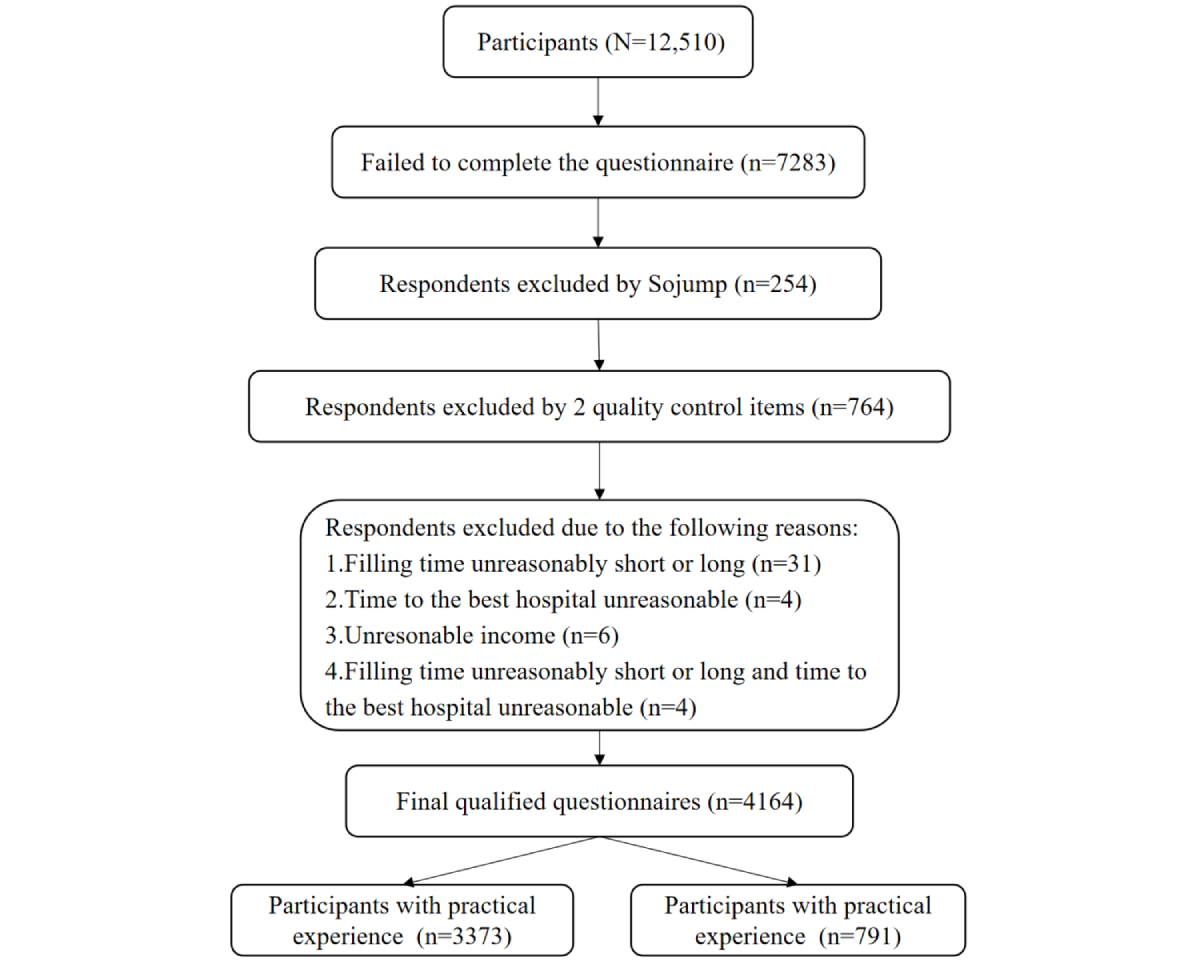
Sampling procedure.

In addition, data were screened based on 3 basic information, namely, filling time, income, and time to visit the nearest best hospital. The mean filling time was 409 (SD 401.264) seconds. A total of 35 questionnaires were excluded because their filling time was more than the mean + 3 SDs or less than the mean – 3 SDs. There were 6 questionnaire responses in which an income information choice was extremely unreasonable. There were 8 questionnaire responses in which a choice on time to arrive at the best hospital was extremely unreasonable. There were 10 questionnaire responses with unreasonable choices on time to arrive at the hospital and income. The survey was conducted from December 24, 2020, to January 14, 2021. A total of 12,510 people visited the questionnaire link, of whom 41.78% (5227/12,510) completed the questionnaire, and 4164 valid questionnaires were included (Figure 4). Among the 4164 valid questionnaires, 791 participants have practical experience with e-consultation for drug eruption.

### Ethical Considerations

This study was approved by the Ethics Committee of Capital Medical University (number Z2019SY017). At the beginning of the electronic questionnaire, the following information was first provided: the purpose of the questionnaire, information and instructions regarding the questionnaire, assurance of proper handling of personal information, and the names of research institutions. All questionnaires were anonymous. The questionnaire link provided on the online questionnaire response collection platform cannot be copied. After users complete the questionnaire through the link, the link will be removed from the list and cannot be used repeatedly.

### Data Analysis

#### Overview

The data of this study are divided into 2 parts for analysis: 3373 participants without any practical experience with e-consultation for drug eruption and 791 with practical experience. The data on participants without practical experience were used to perform analysis on the geographical characteristics, perception of disease severity, perceived probability of curing the disease, patients’ selection of treatment, patients’ selection of hospitals, patients’ selection of doctors, and logistic regression analysis of treatment selection in the different scenarios. The participants were randomly grouped, and so we analyzed the differences in outcome between different groups under different scenarios. The data on participants with practical experience were used to perform an analysis on the medical judgment and choices of patients with real drug eruption. The data analysis methods adopted in this study are described in the following sections.

#### Test of Normality

The normality of the outcomes affected the choice of statistics test. In this study, SPSS 20.0 (IBM Inc) was used to first conduct the Kolmogorov-Smirnov test and Shapiro-Wilk test on the data.

#### Rank Sum Test

After the normality test, it was found that the collected data were skewed, and a rank sum test was adopted to test the differences in outcome except for the enumeration data. In both scenarios 1 and 2, each participant performed a self-control study; therefore, the paired Wilcoxon signed rank test was selected to test the self-control outcomes. As for the other independent outcomes of the 4 scenarios, the Mann-Whitney U test was performed.

#### Chi-Square Test

Some outcomes such as medical treatment selection were enumeration data, so the chi-square test was adopted. In both scenarios 1 and 2, there were self-control outcomes, so the McNemar test was used. As for the other independent outcomes, the chi-square test was performed.

#### Logistic Regression

Although the scenarios were assigned randomly to the participants, the statistical test showed that the distribution of participants’ characteristics had significant (*χ*^2^_6_ [residence]=35.892, *P*<.001; *χ*^2^_12_ [education]=53.657, *P*<.001; *χ*^2^_9_ [income]=33.786, *P*<.001; *χ*^2^_9_ [risk attitude score]=19.361, *P*=.02) differences in each scenario, which means that the characteristic influence of each scenario was not eliminated by the random effect. Therefore, the characteristics may result in significant differences in the patients’ medical selection. To ensure that the result was robust, characteristics were taken as control variables and medical selection before and after e-consultation was taken as the dependent variable in the logistic regression model for analysis. In the logistic regression model, the independent variable can be either categorical or continuous. However, it was shown that if a continuous variable was directly taken into the model, the results of the model may miss related factors [35]. Therefore, all independent variables were converted into categorical variables.

## Results

### Summary Statistics

The descriptive statistics of the participants without practical experience are presented in Table 2. In this survey, there was no noticeable difference in geographical characteristics distribution among the total sample and 4 scenarios. Overall, the distribution of gender was balanced. Of the total respondents, 78.2% (2604/3373) were younger than 40 years, 64.9% (2189/3373) had a bachelor’s degree or higher, 74.2% (2501/3373) were from urban areas, 15.8% (533/3367) took more than 30 minutes to arrive at the best hospitals, 30.7% (1035/3367) took less than 10 minutes, and 32.2% (1086/3367) took 11-20 minutes. The income of the respondents was distributed at all levels. Only 1.9% (63/3373) of the respondents had no medical insurance, and 60.6% (2044/3373) had urban employee medical insurance. The proportion of respondents whose health literacy score was 11-15 points accounted for 27.6% (932/3373) of the total sample, while those whose health literacy scores were 10 and 9 accounted for 25.9% (873/3373) and 20.9% (704/3373), respectively. The risk attitude score of 75.8% (2558/3373) of the respondents was higher than 24.

**Table 2:** Geographical characteristics of the respondents (N=3373).

Characteristics		Total sample (n=3373), n (%)	Scenario 1 (n=740), n (%)	Scenario 2 (n=913), n (%)	Scenario 3 (n=775), n (%)	Scenario 4 (n=945), n (%)
**Gender**						
	Male	1591 (47.2)	366 (49.5)	429 (47.0)	351 (45.3)	445 (47.1)
	Female	1782 (58.8)	374 (50.5)	484 (53.0)	424 (54.7)	500 (52.9)
**Age (years)**						
	18-25	874 (25.9)	186 (25.1)	246 (26.9)	197 (25.4)	245 (25.9)
	26-30	671 (19.9)	153 (20.7)	165 (18.1)	175 (22.6)	178 (18.8)
	31-40	1086 (32.2)	235 (31.8)	289 (31.7)	243 (31.4)	319 (33.8)
	41-50	561 (16.6)	130 (17.6)	158 (17.3)	119 (15.4)	154 (16.3)
	51-60	158 (4.7)	29 (3.9)	49 (5.4)	35 (4.5)	45 (4.8)
	≥61	23 (0.6)	7 (0.9)	6 (0.7)	6 (0.8)	4 (0.4)
**Education**						
	Middle school or lower	236 (7.0)	32 (4.3)	87 (9.5)	36 (4.6)	81 (8.6)
	High school	368 (10.9)	63 (8.5)	118 (12.9)	69 (8.89)	118 (12.5)
	Three-year college	580 (17.2)	127 (17.2)	143 (15.7)	130 (16.8)	180 (19.0)
	Bachelor	1935 (57.4)	452 (61.1)	500 (54.8)	474 (61.4)	509 (53.9)
	Master or higher	254 (7.5)	66 (8.9)	65 (7.1)	66 (8.5)	57 (6.0)
**Residence**						
	Rural	486 (14.4)	76 (10.3)	153 (16.7)	84 (10.8)	173 (18.3)
	Suburban	386 (11.4)	81 (10.9)	100 (11.0)	92 (11.9)	113 (12.0)
	Urban	2501 (74.2)	583 (78.8)	660 (72.3)	599 (77.3)	659 (69.7)
**Income in 2019 (RMB)^a^**						
	0-8	972 (29.1)	166 (22.6)	280 (30.9)	208 (27.0)	318 (33.9)
	9-15	1116 (32.1)	252 (34.4)	298 (32.9)	265 (34.5)	301 (32.1)
	16-20	564 (17.2)	145 (19.8)	137 (15.1)	143 (18.6)	139 (14.8)
	>21	692 (20.7)	170 (23.2)	190 (21.0)	153 (19.9)	179 (19.1)
	Missing value	29 (0.8)	7 (0.9)	8 (0.8)	6 (0.8)	8 (0.8)
**Time to the best hospital (minutes)^b^**						
	0-10	1035 (30.7)	231 (31.3)	293 (32.1)	213 (27.5)	298 (31.7)
	11-20	1086 (32.3)	246 (33.3)	284 (31.1)	264 (34.1)	292 (31.0)
	21-30	713 (21.2)	152 (20.6)	190 (20.8)	176 (22.7)	195 (20.7)
	>31	533 (15.8)	110 (14.9)	145 (15.9)	122 (15.7)	156 (16.6)
	Missing value	6 (0.2)	1 (0.1)	1 (0.1)	0 (0)	4 (0.4)
**Insurance**						
	UEMI^c^	2044 (60.6)	488 (65.9)	529 (57.9)	488 (63.0)	539 (57.0)
	BMIURR^d^	1445 (42.8)	283 (38.2)	422 (46.2)	294 (37.9)	446 (47.2)
	Free medical care	256 (7.6)	60 (8.1)	77 (8.4)	47 (6.1)	72 (7.6)
	CMI^e^	714 (21.2)	188 (25.4)	172 (18.8)	172 (22.2)	182 (19.3)
	None	63 (1.9)	13 (1.8)	19 (2.1)	16 (2.1)	15 (1.6)
**Health literacy score**						
	3-8	864 (25.6)	184 (24.9)	236 (25.8)	195 (25.2)	249 (26.3)
	9	704 (20.9)	150 (20.3)	201 (22.0)	178 (23.0)	175 (18.5)
	10	873 (25.9)	212 (28.6)	221 (24.2)	191 (24.6)	249 (26.3)
	11-15	932 (27.6)	194 (26.2)	255 (27.9)	211 (27.2)	272 (28.8)
**Risk attitude score**						
	6-23	815 (24.2)	160 (21.6)	236 (25.8)	178 (23.0)	241 (25.5)
	24-25	855 (25.3)	195 (26.4)	237 (26.0)	176 (22.7)	247 (26.1)
	26-27	1102 (32.7)	263 (35.5)	279 (30.6)	253 (32.6)	307 (32.5)
	28-30	601 (17.8)	122 (16.5)	161 (17.6)	168 (21.7)	150 (15.9)

^a^US $1=6.475 RMB.

^b^Measures the accessibility of high-quality medical resources.

^c^UEMI: urban employee medical insurance.

^d^BMIURR: basic medical insurance for urban and rural residents.

^e^CMI: commercial medical insurance.

### Perception of Disease and Medical Institution

As shown in Table 3, in scenario 1 (mild cases), only 14.1% (104/740) of the 740 participants considered their diseases as not serious before they used e-consultation. By contrast, after e-consultation, 69.5% (539/775) of the participants considered their diseases as not serious. The Mann-Whitney U test showed that the differences in the perception of disease severity before and after e-consultation were significant (*P*<.001). The same conclusion was reached after the Wilcoxon matched-pairs signed rank test of the results of scenario 1. It can be observed that the differences in disease perception before and after e-consultation were not caused by the order of questionnaire design. In scenario 2 (severe cases), before e-consultation, 54.1% (494/913) of the 913 participants considered their diseases as very serious, but after e-consultation, only 16.6% (157/945) considered their diseases as very serious. The same result was obtained after the 2 aforementioned tests as well.

On the whole, participants’ perception of medical institutions’ capability has gradually increased with the promotion of institutions’ level, as shown in Table 4. In the mild cases, the median cure probability in tertiary hospitals reached 95%. After e-consultation, participants’ perception of the curing capability of various medical institutions had significant (*P*<.001) improvement. In the severe cases, after e-consultation, participants’ perception of the curing capability of nontertiary hospitals decreased (self-contrast results in scenario 2) or did not significantly (for self-treatment, Z=–1.721, *P*=.09; for community health center, Z=–0.928, *P*=.35; for district/county hospital, *P*=.34) change (comparison between scenarios 2 and 4), whereas participants’ perception of the curing capability of the tertiary hospital was significantly (*P*=.01) improved.

**Table 3:** Perception of disease severity.

Scenario		Not at all, n (%)	Not serious, n (%)	Unclear, n (%)	Serious, n (%)	Very serious, n (%)	Z (*P* value)
**Scenario 1 (n=740)**							−23.393 (<.001)
	Before	10 (1.4)	104 (14.1)	176 (23.8)	410 (55.4)	40 (5.4)	
	After	18 (2.4)	540 (73.0)	98 (13.2)	82 (11.1)	2 (0.3)	
**Scenario 3 (n=775)**							−23.534 (<.001)
	After	17 (2.2)	539 (69.5)	152 (19.6)	62 (8.0)	5 (0.6)	
**Scenario 2 (n=913)**							−14.233 (<.001)
	Before	7 (0.8)	4 (0.4)	46 (5.0)	362 (39.6)	494 (54.1)	
	After	4 (0.4)	18 (2.0)	111 (12.2)	558 (61.1)	222 (24.3)	
**Scenario 4 (n=945)**							−17.108 (<.001)
	After	1 (0.1)	24 (2.5)	166 (17.6)	597 (63.2)	157 (16.6)	

**Table 4:** Perceived probability of curing the disease.

Scenario			Self-treatment		Community health center		District/county hospital		Tertiary hospital	
			P^a^ (IQR), n	Z (*P* value)	P (IQR), n	Z (*P* value)	P (IQR), n	Z (*P* value)	P (IQR), n	Z (*P* value)
**Scenario 1**				−19.934 (<.001)		−15.516 (<.001)		−11.994 (<.001)		−6.711 (<.001)
	Before		40 (21-60)		63 (45-79)		80 (67-89)		95 (87-100)	
	After		70 (53-81)		78 (61-88)		86 (75-96)		98 (90-100)	
**Scenario 3**				−18.458 (<.001)		−11.091 (<.001)		−8.417 (<.001)		−2.863 (.004)
	After		69 (57-80)		77 (63-85)		86 (78-95)		97 (90-100)	
**Scenario 2**				−5.426 (<.001)		−5.597 (<.001)		−4.676 (<.001)		−3.971 (<.001)
	Before		20 (6-40)		42 (28-61)		66 (52-80)		90 (80-99)	
	After		16 (5-31)		40 (24-59)		64 (49-80)		91 (80-100)	
**Scenario 4**				−1.721 (.09)		−.928 (.35)		−.962 (.34)		−2.467 (.01)
	After		21 (10-34)		44 (30-60)		68 (55-80)		91 (81-100)	

^a^Perceived probability.

### Intention of Medical Selection

As shown in Tables 5 and 6, in the mild cases, 56.6% (419/740) of the participants chose to directly visit the hospital before e-consultation. After e-consultation, the online doctors provided patients with self-treatment suggestions, and 71.4% (553/775) of the participants chose self-treatment. The chi-square test determined that there was a significant (*P*<.001) difference in the medical choice before and after e-consultation. In the severe cases, before e-consultation, 92.8% (847/913) of the participants chose to directly visit the hospital. After e-consultation, the doctors suggested that the patients should visit the hospital directly, and 94.9% (897/945) of the participants chose to visit the hospital. The chi-square test showed that there were no significant (*P*=.05) differences. Additionally, the McNemar test determined that the results of parallel contrast groups (scenarios 1 and 3/scenarios 2 and 4) were the same.

Of the participants who chose to directly visit the hospital, before e-consultation, 50.4% (211/419) chose tertiary hospitals for medical treatment in the mild cases. After e-consultation, 47.0% (85/181) of the participants chose a tertiary hospital, and among them, 4.5% (10/222) wanted to visit the hospital where the online doctors worked. In the severe cases, before e-consultation, 66.7% (565/847) of the participants chose tertiary hospitals. After e-consultation, 64.2% (576/897) chose tertiary hospitals, and among them, 11.3% (101/897) wanted to visit the hospital where the online doctors worked. In both mild and severe cases, after e-consultation, the proportion of participants who chose primary health institutions gradually decreased. See Table 7 for detailed data.

In the mild cases, before e-consultation, 419 participants chose to directly visit the hospital, of whom 53.0% (222/419) would select specialists. After e-consultation, 222 participants chose to directly visit the hospital, of whom 25.7% (57/222) wanted to consult the online doctors face-to-face and 53.2% (118/222) wanted to consult specialists. In the severe cases, before e-consultation, 847 participants chose to directly visit the hospital, of whom 75.3% (638/847) wanted to consult specialists. After e-consultation, 897 participants chose to visit the hospital, of whom 56.4% (506/897) wanted to consult the online doctors face-to-face and 33.6% (301/897) wanted to consult specialists. See Table 8 for detailed data.

**Table 5:** Patients’ selection of treatment (group comparison).

Scenario	Self-treatment, n (%)	Direct hospital visit, n (%)
Scenario 1^a^ (n=740; before)	321 (43.4)	419 (56.6)
Scenario 3^a^ (n=775; after)	553 (71.4)	222 (28.6)
Scenario 2^b^ (n=913; before)	66 (7.2)	847 (92.8)
Scenario 4^b^ (n=945; after)	48 (5.1)	897 (94.9)

^a^For scenarios 1 and 3, the *χ*^2^ (*df*) and *P* values are 121.384 (1) and <.001, respectively.

^b^For scenarios 2 and 4, the *χ*^2^ (*df*) and *P* values are 3.726 (1) and .05, respectively.

**Table 6:** Patients’ selection of treatment (self-control comparison).

Before		After		*χ*^2^/*df* (*P* value)
		Self-treatment	Direct hospital visit	
**Scenario 1**				163.711/1 (<.001)
	Self-treatment (n=321)	267/559	54/181	
	Direct hospital visit (n=419)	292/559	127/181	
**Scenario 2**				1.742/1 (.22)
	Self-treatment (n=66)	11/53	55/860	
	Direct hospital visit (n=847)	42/53	805/860	

**Table 7:** Patients’ selection of hospitals.

Scenario		Online doctor’s hospital, n (%)	Local tertiary hospital, n (%)	Local district/county hospitals, n (%)	Local community health center, n (%)	Hospitals in other cities, n (%)
**Scenario 1**						
	Before (n=419)	N/A^a^	211 (50.4)	158 (37.7)	50 (11.9)	0 (0)
	After (n=181)	10 (5.5)	85 (47.0)	67 (37.0)	19 (10.5)	0 (0)
**Scenario 3**						
	After (n=222)	10 (4.5)	117 (52.7)	77 (34.7)	17 (7.7)	1 (0.5)
**Scenario 2**						
	Before (n=847)	N/A	565 (66.7)	223 (26.3)	54 (6.4)	5 (0.6)
	After (n=860)	105 (12.2)	566 (65.8)	159 (18.5)	25 (2.9)	5 (0.6)
**Scenario 4**						
	After (n=897)	101 (11.3)	576 (64.2)	190 (21.2)	29 (3.2)	1 (0.1)

^a^N/A: not applicable.

**Table 8:** Patients’ selection of doctors.

Types of doctors	Scenario 1			Scenario 3	Scenario 2		Scenario 4
	Before (n=419), n (%)	After (n=181), n (%)	After (n=222), n (%)		Before (n=847), n (%)	After (n=860), n (%)	After (n=897), n (%)
The consulted doctor	N/A^a^	59 (32.6)	57 (25.7)		N/A	483 (56.2)	506 (56.4)
Specialist	222 (53.0)	70 (38.7)	118 (53.2)		638 (75.3)	272 (31.6)	301 (33.6)
General practitioner	197 (47.0)	52 (28.7)	47 (21.2)		209 (24.7)	105 (12.2)	90 (10.0)

^a^N/A: not applicable.

### Robustness Test

To exclude bias, the characteristics were used as control variables in the logistic regression model. In scenarios 1 and 2, patients made treatment selection before e-consultation. This can reflect the selection without e-consultation. Therefore, the dependent variables in the regression model were the treatment selection before e-consultation in scenarios 1 and 2 and the treatment selection after e-consultation in scenarios 3 and 4. The independent variables included personal characteristics, the use of e-consultation, and the severity of the disease (mild case=0, severe case=1).

As shown in Table 9, logistic regression results showed that risk attitude (for 24-25, odds ratio [OR] 1.393, *P*=.02; for 26-27, OR 1.732, *P*<.001; for 28-30, OR 2.219, *P*<.001), e-consultation (for did not use, OR 0.428, *P*<.001), and the severity of disease (for serious, OR 24.994, *P*<.001) were significant factors affecting the choice of medical treatment. It could be seen that although the results of medical selection in this study have been affected by risk attitude, the 2 most significant factors were research intervention and scenarios classification standard. Compared with patients who did not utilize e-consultation, those using e-consultation were significantly less willing to directly visit the hospital (OR 0.428, *P*<.001).

**Table 9:** Logistic regression analysis of treatment selection.

Characteristics		Odds ratio (95% CI)	*P* value
**Gender**			
	Male	1.000	
	Female	1.008 (0.828-1.226)	.94
**Age (years)**			
	18-25	1.000	
	26-30	0.939 (0.707-1.247)	.66
	31-40	0.725 (0.558-0.941)	.02
	>41	0.881 (0.657-1.181)	.40
**Education**			
	High school or lower	1.000	
	3-year college	0.878 (0.618-1.247)	.47
	Bachelor’s degree	0.715 (0.523-0.979)	.04
	Master’s degree or higher	0.654 (0.419-1.020)	.06
**Residence**			
	Nonurban	1.000	
	Urban	0.955 (0.745-1.225)	.72
**Income in 2019 (RMB^a^)**			
	0-8	1.000	
	9-15	1.064 (0.821-1.380)	.64
	16-20	0.969 (0.708-1.325)	.84
	>21	1.022 (0.750-1.391)	.89
**Time to the best hospital (minutes)^b^**			
	0-10	1.000	
	11-20	1.092 (0.862-1.385)	.47
	21-30	0.779 (0.597-1.016)	.07
	>31	1.054 (0.780-1.424)	.73
**Health insurance**			
	With	1.000	
	Without	1.229 (0.607-2.487)	.57
**Health literacy score**			
	3-8	1.000	
	9	0.928 (0.704-1.222)	.59
	10	0.998 (0.769-1.296)	.99
	11-15	0.847 (0.653-1.099)	.21
**Risk attitude**			
	6-23	1.000	
	24-25	1.393 (1.064-1.822)	.02
	26-27	1.732 (1.341-2.237)	<.001
	28-30	2.129 (1.576-2.875)	<.001
**Disease severity**			
	Mild	1.000	
	Serious	24.994 (19.859-31.457)	<.001
**Use of e-consultation**			
	Used	1.000	
	Did not use	0.428 (0.354-0.518)	<.001

^a^US $1=6.475 RMB.

^b^The question of “Time to the best hospital” is as follows: “How fast does it take to get from your home to the best hospital in your district/county?”

### Practical Experience With e-Consultation for Drug Eruption

As shown in Table 10, a total of 791 participants had practical experience with e-consultation for drug eruption: a total of 332 participants received suggestions for self-treatment, and 459 participants were advised to visit the hospital directly. With regard to the suggestions for self-treatment and visiting the hospital, 79.5% (264/332) and 93.5% (429/459) of the participants trusted the doctors’ judgments, respectively. Of those with less trust in doctors’ suggestion for self-treatment, 56% (38/68) still chose to be treated in a local tertiary hospital, and 41% (28/68) wanted to consult their online doctors face-to-face. Of those who followed the doctors’ suggestions that they should be treated in a hospital directly, 57.8% (248/429) chose to visit a local tertiary hospital, and 54.3% (233/429) wanted to consult the online doctors face-to-face. The selection of treatment, hospital, and doctor by participants with practical experience was highly consistent with the results of the scenario survey.

**Table 10:** Investigation of patients with practical e-consulting experience.

Medical attitude and selection		Self-treatment, n/N (%)	Treated in hospital directly, n/N (%)
**Attitude to judgment**			
	Trust	264/332 (79.5)	429/459 (93.5)
	Distrust	68/332 (20.5)	30/459 (6.5)
**Selection of hospital**			
	Online doctor’s hospital	7/68 (10.3)	98/429 (22.8)
	Local tertiary hospital	38/68 (55.9)	248/429 (57.8)
	Local district/county hospitals	17/68 (25.0)	71/429 (16.6)
	Local community health center	6/68 (8.8)	9/429 (2.1)
	Hospitals in other cities	0/68 (0.0)	3/429 (0.7)
**Selection of doctor**			
	The consulted doctor	28/68 (41.2)	233/429 (54.3)
	Specialist	31/68 (45.6)	135/429 (31.5)
	General practitioner	9/68 (13.2)	61/429 (14.2)

## Discussion

### Principal Findings

#### After E-consultation, Patients’ Evaluation of Diseases and Medical Institutions Changed

Advantageous selection theory holds that the risk perception can adjust the adverse selection caused by information asymmetry. Patients’ perception of diseases is the reflection of risk perception in medical selection. The results showed that before e-consultation, 55.4% (410/740) and 5.4% (40/740) of the participants with mild diseases considered their diseases as “serious” and “very serious,” respectively, but after e-consultation, these rates decreased to 11.1% (82/740) and 0.3% (2/740), respectively. In the severe cases, before e-consultation, 39.6% (362/913) and 54.1% (494/913) of the participants considered their diseases as “serious” and “very serious,” respectively, but after e-consultation, these rates changed to 61.1% (558/913) and 24.3% (222/913), respectively. This indicates that the judgments of online doctors can be effectively accepted by the patients and that patients’ understanding of the disease has been improved. Patients’ acceptance of the suggestion also means that the information asymmetry of patients was decreased after e-consultation, which was consistent with the conclusion of a previous study [36]. It can be observed that just increasing the amount of information cannot effectively improve the patients’ information level. Only when the information source provides the patients with comprehensible health information can the patients receive satisfactory information [37].

With regard to medical institutions’ capability, it was found that after e-consultation, patients have a more rational understanding of the capability. Participants with mild diseases believed that the probability that medical institutions could cure their diseases was significantly improved because they realized that their diseases were not serious. Additionally, for those patients with serious diseases, their capability assessment of low-level medical institutions was decreased because online doctors recommended them to visit the hospital, which made them think that their diseases were very serious. Therefore, they preferred to choose the tertiary hospital and regarded other low-level medical institutions as incapable of treating serious diseases. Disease severity and evaluation of medical institutions are important factors affecting medical selection. The results of this study showed that the 2 factors have changed significantly after e-consultation, which allowed us to better understand how e-consultation affects medical selection. A qualitative study could be conducted in the future to better understand the influence mechanism of these factors on their decision making.

#### E-consultation Is Expected to Guide Patients to Seek Reasonable Medical Services

Before e-consultation, 56.6% (419/740) of the participants chose to directly visit the hospital. After e-consultation, 71.4% (553/775) of the participants had confidence in doctors’ judgments and wanted to conduct self-treatment. It can be observed that after e-consultation, there were significant changes in the medical choice for patients with mild diseases. For patients with serious diseases, after e-consultation, the intention to directly visit the hospital strengthened. Our results indicate that online medical suggestion was accepted by patients and had a real influence on their medical selection. Based on the expected utility and prospect theories, if patients were reassessed on their disease severity and the capability of medical institutions, they would change their expected utility of hospitals and doctors [38]. The results of this study verified this theory, because patients’ medical selection and evaluation of diseases were more consistent with online doctors’ judgment after e-consultation. One possible reason is that patients found low-level hospitals also could meet their medical demands after e-consultation, which made the flow direction of patients reach a separating equilibrium. Specifically, patients with mild diseases took self-treatment or went to primary medical institutions for medical treatment, whereas those with severe diseases went directly to hospitals, rather than all patients entering directly into the high-level hospital.

The reduction of information asymmetry between doctors and patients also has positive impacts on offline medical services. The information gap between disease and drug will cause patients to distrust doctors and even lead to aggression [39]. E-consultation can narrow this information gap before face-to-face consultation. Moreover, e-consultation enables patients to participate in medical decision making, which is expected to improve patient satisfaction and strengthen the trust between doctors and patients [40,41]. In addition, there are no recognizable differences in service quality, satisfaction, and interpersonal relationship established between e-consultation and face-to-face consultation [42]. Therefore, the use of e-consultation within the offline medical system can effectively improve the information level of patients, the degree of trust between doctors and patients, and the quality of medical services. The doctors’ response time to the patients, service attitude, and other factors have a significant effect on the patients’ acceptance of e-consultation [43].

However, in e-consultation, patients are guided to assess the disease reasonably, but that does not mean that patients can make the best selection. Online doctors only suggest that patients be treated in hospitals, not which hospitals to visit. Additionally, patients still prefer to choose a tertiary hospital. Notably, in China, the hospital information disclosure system is underdeveloped. Patients do not know the characteristics, specializations, costs, and other conditions of various medical institutions. Perhaps, the combination of e-consultation and information disclosure can better guide patients to make more reasonable selection of hospitals and doctors [44]. When disclosing hospital information, the suitability of the information should also be considered. Even when the patients are informed about various detailed information on the hospital, they will still experience difficulties in making an ideal choice owing to a lack of health literacy [45].

#### E-consultation May Become a New Means for Doctors to Attract Patients

Some studies have indicated that if medical services are transformed from offline to online settings, then the services will become more centralized [46]. This study also found that during offline consultation, many patients still preferred to consult the same online doctors who provided e-consultation. The reasons for this situation may be attributed to 2 aspects: first, most online doctors are from high-level hospitals, leading patients to believe that these doctors have high-level medical abilities; second, the doctors consulted online are trusted better because they are more familiar with patients’ illnesses after e-consultation, although their modes of communication were only through simple texts and images. When patients insist in seeking face-to-face medical consultation, doctors who performed e-consultation previously know better about their disease. Moreover, patients’ medical decision making is not solely dependent on online doctor-patient communication. Doctors giving e-consultations are from hospitals across the country, and so patients will have to incur many indirect costs to consult the online doctors face-to-face. However, the disconnect between intention and selection signifies that e-consultation can indeed overcome the shortage of offline medical resources.

In addition, this study did not explore the influence when suggestions during e-consultation were incorrect. Based on the study result that most patients chose to trust online doctors, it may be deduced that these patients will also choose to believe incorrect suggestions. Of course, the influence of incorrect e-consultation suggestion needs more research. This also reminds us that medical quality control in e-consultation is necessary; otherwise, these errors will cause medical disputes, mismanagement of medical resources, and other medical problems, which will lead to a decrease in willingness to use e-consultation. Further, the quantity of high-quality online doctors remains limited. Thus, the imbalance between supply and demand of e-consultation will cause adverse selection and moral hazard for online doctors [47], which will greatly reduce the service quality and lead patients to revert to offline consultation. Therefore, research on rational allocation and utilization of e-consultation needs to be conducted in the future.

### Limitations

This research was conducted using a scenario survey, and thus it lacks the type of real-world data generated during a field survey. Moreover, participants in the survey cannot be considered a true match to real-world patients. Hence, the results of a scenario survey may be slightly different from medical selection in the real world. Additionally, only drug eruption was selected in the scenario survey of this study. The results of this study are only applicable to common e-consulting diseases. Although this disease is highly representative, the application of the research results will need to be combined with that of specific diseases, especially for uncommon diseases. Ultimately, this study only simulated the correct judgments and diagnoses in e-consultation, and the incorrect suggestions were not considered. Therefore, the impact of incorrect suggestions needs further research. Additional research should be carried out based on the long follow-up data of real patients with e-consultation experience, which should also pay attention to the situation of revisiting patients, misdiagnosis of e-consultation, and other kinds of disease.

### Conclusions

In e-consultation, online doctors’ suggestions and judgments can be effectively accepted by patients and can significantly affect patients’ assessment of diseases, service quality of medical institutions, and medical selection. E-consultation is expected to guide patients to make more reasonable medical choices. However, patients also need adequate information from the hospital to achieve a reasonable match between disease and hospital. Therefore, it is difficult for e-consultation to play a complete role in the guidance of medical selection independently without the help of other systems conducive to reasonable patient choice. In addition, there may be a significant patient concentration effect in the e-consultation market if governments do not regulate it reasonably.

## Abbreviations

BHLS: Brief Health Literacy Screen
BMIURR: basic medical insurance for urban and rural residents
CMI: commercial medical insurance
DOSPERT: Domain-Specific Risk-Taking
OR: odds ratio
UEMI: urban employee medical insurance

## Data Availability

The data sets analyzed during this study are available from the corresponding author on reasonable request.
